# Nonparametric estimation of a primary care production function in urban Brazil

**DOI:** 10.1186/s13561-020-00294-9

**Published:** 2020-11-28

**Authors:** Bruno Wichmann, Roberta Wichmann

**Affiliations:** 1 Department of Resource Economics and Environmental Sociology, University of Alberta, 515 General Services Building, Edmonton, AB T6G 2H1 Canada; 2 World Bank, SCN Quadra 2, Lote A, Ed. Corporate Financial Center, 7o Andar, Brasília, DF CEP 70712-900 Brazil; 3 Department of Public Health University of Brasília, Brasília, DF Brazil

**Keywords:** Primary care, Public healthcare investment, Returns to capital and labor, Heterogeneity, Nonlinearities, Complementarities

## Abstract

**Background:**

The Brazilian public health system is one of the largest health systems in the world, with a mandate to deliver medical care to more than 200 million Brazilians. The objective of this study is to estimate a production function for primary care in urban Brazil. Our goal is to use flexible estimates to identify heterogeneous returns and complementarities between medical capital and labor.

**Methods:**

We use a large dataset from 2012 to 2016 (with more than 400 million consultations, 270 thousand physicians, and 11 thousand clinics) to nonparametrically estimate a primary care production function and calculate the elasticity of doctors’ visits (output) to two inputs: capital stock (number of clinics) and labor (number of physicians). We benchmark our nonparametric estimates against estimates of a Cobb-Douglas (CD) production function. The CD model was chosen as a baseline because it is arguably the most popular parametric production function model. By comparing our nonparametric results with those from the CD model, our paper shed some light on the limitations of the parametric approach, and on the novelty of nonparametric insights.

**Results:**

The nonparametric results show significantly heterogeneity of returns to both capital and labor, depending on the scale of operation. We find that diseconomies of scale, diminishing returns to scale, and increasing returns to scale are possible, depending on the input range.

**Conclusions:**

The nonparametric model identifies complementarities between capital and labor, which is essential in designing efficient policy interventions. For example, we find that the response of primary care consultations to labor is steeper when capital level is high. This means that, if the goal is to allocate labor to maximize increases in consultations, adding physicians in cities with a high number of clinics is preferred to allocating physicians to low medical infrastructure municipalities. The results highlight how the CD model hides useful policy information by not accounting for the heterogeneity in the data.

## Background

Primary health care represents a broad approach to the promotion of individual and societal health and well-being, and as such it includes health services employed to deliver prevention, treatment, rehabilitation, and palliative care. According to the World Health Organization (WHO), the delivery of quality primary care can have significant short-term impacts in reducing risk factors and poor health conditions [1]. The WHO recognizes that health systems based on primary health care are of paramount importance in achieving sustainable health goals. This architecture is especially important in developing countries, where primary care systems often need to be further developed. WHO works with many countries to implement primary health care policies that integrate health-promoting and preventive interventions thereby reducing health care delivery costs and improving efficiency through lower hospital admissions.

Many developing and middle-income countries have been struggling to maintain adequate levels of public services in light of increasingly tighter budget constraints. For example, since 2010, Latin American countries have experienced declining GDP per capita growth rates, which dropped from 4.67% to − 0.44% in 2018. In fact, the region has been averaging a negative growth rate since 2014 [2]. This trend highlights the struggle of financing improvements in standards of living when wealth grows at a rate slower than that of population. A study by Varela and co-authors shows that only 6% of the municipalities in Sao Paulo, the largest Brazilian state, efficiently allocate health care expenditures to the delivery of primary care [3]. Lobo et al. examine a sample of 104 Brazilian teaching hospitals finding similar results; only 5% of the hospitals efficiently allocate resources [4].

As population grows, so does the demand for health care. In this context, it is essential for managers and policy makers to carefully understand the determinants of medical services uptake. One approach is to estimate health care production as a function of capital infrastructure in the healthcare system and the availability of health professionals. Such estimates allow for an evaluation of the returns to investments in capital and labor. Moreover, it is possible, and even probable, that the outcomes of health care investments are nonlinear, and the impact of health policies may vary depending on the scale of capital and labor. For example, it is possible for investments on medical personnel to have differentiated impacts on output as we move along the distribution of both labor and capital. Are returns to labor higher when labor is low? Are returns to labor investments a function of the stock of capital? Having an approach to answer these types of questions is imperative for the efficient design of health policy.

The goal of this paper is to estimate a primary care production function for urban Brazil. We employ nonparametric methods to estimate the elasticities of the uptake of primary health care services to capital infrastructure (number of clinics) and labor (number of physicians). We contrast our approach with a popular parametric baseline model to highlight the advantages of nonparametric approaches, and to show how the more flexible nonparametric estimates can provide additional support to evidence-informed public policies.

### Literature and contributions

Examination of health production using parametric and nonparametric approaches has been an ongoing and fruitful area of research in the health economics and policy fields. The literature is large and evolved in many directions. For example, the efficiency of health care delivery systems has been studied using parametric models such as the Stochastic Frontier Approach [5–7] and nonparametric models such as Data Envelopment Analysis [8–10]. Some papers explore the benefits of parametric estimation trying to limit the disadvantages of the parametric assumption by using flexible functional forms to estimate primary care or health production functions [11, 12]. Other papers examine how the choice of functional form affect parametric estimation of health care technical efficiency [13]. Health economics research has also compared parametric and nonparametric methods [14, 15].

We add to this literature by using both parametric and nonparametric approaches to estimate elasticities of primary health care delivery in a large urban setting: the Brazilian public health system. Our contribution is twofold. First, by studying primary care in Brazil we are examining one of the largest public health systems in the world, with a mandate to deliver universal health care free of charges to more than 200 million individuals. We examine the five-year period from 2012 to 2016 where Brazil faced unfavorable economic conditions. As the country (and many others in the region) continues to struggle to revert the economic environment, data-driven and evidence-based policy recommendations are in high demand.

Second, we contribute by constrasting simple parametric estimates with a set of rich estimates from nonparametric local-linear models. Our application takes advantage of large datasets (with millions of observations) to mitigate issues related to the curse of the dimenssionality (although at the expense of computing time). Our paper highlights the insights that nonparametric estimates are able to deliver regarding heterogeneity, nonlinearities, and cross-effects. In doing so, the paper showcases the potential of local-linear models in informing public health policy.

### The Brazilian health system

The Brazilian Unified Health System (Sistema Único de Saúde – SUS) is a public health system created in 1989 that offers access to health care, free of charge, to all Brazilians. SUS serves a population of more than 200 million people, ranking among the largest government funded and managed health care systems of the world. SUS network is responsible for all levels of health care, including all levels of medical care, laboratory and diagnostic care, physical and occupational therapy, nutritional support, pharmaceutical care, etc.

While all Brazilians are covered by SUS, private medical services and health insurances are also available for purchase in Brazil. Table 1 shows total Brazilian population and the number of individuals with supplemental private medical insurance. The data show that Brazilians increasingly depend on publicly provided health care. In the 2012–2016 period, population growth paired with a decrease in private insurance enrolment led to the coverage of the Brazilian population with supplemental private health insurance to decrease from 15.1 to 12.8%.

**Table 1:** Brazilian Population with Private Medical Insurance

Year	Total Population	Private Medical Insurance	Private Coverage
2012	193,946,886	29,307,255	15.1%
2013	201,032,714	29,929,896	14.9%
2014	202,768,562	30,449,912	15.0%
2015	204,450,049	28,502,442	13.9%
2016	206,081,432	26,500,327	12.8%

Source: ANS (www.ans.gov.br/perfil-do-setor/dados-gerais) and IBGE (www.ibge.gov.br). Data excludes dental coverage

## Methods

Both parametric and nonparametric approaches can be used to examine health care delivery productivity and technical efficiency. In productivity studies the interest lies mainly on returns to input usage, or elasticities. Such estimates are valuable from a policy perspective as they can guide the allocation of health care resources. In efficiency studies, researchers are interested in measuring how producers deviate from an estimate of the production function, viewed as the state-of-the-art technology frontier. Therefore, efficiency studies are useful in informing policies with a focus on minimizing waste [16].

Regardless of the focus, i.e. production or efficiency, these studies require the estimation of a production function, which begs the question of which method to use. An important methodological decision is choosing between parametric and nonparametric approaches. There is no consensus in the literature and pros and cons have been reported about both methods. Approaches based on parametric functions are simple and can be easily implemented. Under the proper assumptions, parametric approaches have desirable statistical properties (e.g. fast convergence rates, which is important in small samples). Nonparametric approaches are more flexible as no functional form is pre-specified and the shape of the relationship between health output and inputs is determined by the data. On the downside, nonparametric estimators have low convergence rates and require larger amounts of data to deliver estimation errors equivalent to those from correctly specified parametric counterparts [16].

### Model

We conceptualize the delivery of medical services in terms of a medical care production function [17]. We consider the following random production function for healthcare delivery:

1 $$ Y=f\left(K,L\right)+\varepsilon $$

where *Y* is the delivery of medical services (output) and is determined by two components. The first component is deterministic and depends on health care inputs, while the second is a random component. The deterministic portion of output is given by a production function *f* that depends on capital *K* and labor *L*. The random term *ε* captures unobserved determinants of output and is assumed to be zero-mean.

Our goal is to use data on *Y*, *K*, and *L* to estimate the elasticities of primary care delivery with respect to capital and labor, which are determined by, respectively,

$$ \frac{\partial Y}{\partial K}\frac{K}{Y}\ \mathrm{and}\ \frac{\partial Y}{\partial L}\frac{L}{Y} $$.

We are also interested in examining estimates of the conditional mean of output, *E*(*Y*| *K*, *L*). These estimates allow us to visualize partial prediction plots, which can be of great value to policy makers. These types of plots are a simple way to illustrate nonlinearities and, as a result, they help inform policy by highlighting complementarities between capital and labor (see Fig. 2).

### Estimation

Our approach follows closely the production function estimation described by Henderson and Parmeter [18].^1^ We consider two approaches to estimate a cross-city primary care production function in Brazil. The first is a parametric approach based on a Cobb-Douglas production function. The second is a nonparametric approach where no functional form is specified for primary care production.

### Parametric model

This section presents a typically used parametric production model to establish a baseline for comparison with the nonparametric estimates. The Cobb-Douglas production function is arguably the most common parameterization of production in the literature and has been used to model production processes for more than one hundred years [19]. For the case of two inputs, capital and labor, the Cobb-Douglas model with an additively separable error term assumes the primary care function (1) takes the form

$$ Y=A{K}^{\alpha }{L}^{\beta }+\varepsilon $$

where *A* is a technology parameter, *α* is the elasticity of primary care output with respect to medical capital, and *β* is the elasticity of output with respect to the number of physicians. The typical approach to estimate our parameters of interest *α* and *β* is to log-linearize the model and use Ordinary Least Squares (OLS) to estimate the parameters in a log-linear regression. Nevertheless, it has been shown in the literature that such an approach can introduce bias [18, 20]. To avoid such a bias, we estimate *α* and *β* using Nonlinear Least Squares (NLS).

The NLS estimation procedure is as follows. To simplify notation, let *f*(*X*, *θ*) = *AK*^*α*^*L*^*β*^, where *X* = (*K*, *L*) and *θ* = (*A*, *α*, *β*). The nonlinear least squares estimator $\hat{\theta}$ is the value of *θ* that minimizes the sum of the squared residuals [21]:

$$ \underset{\theta }{\min\ }\left(Y-f\left(X,\theta \right)\right)^{\prime}\left(Y-f\left(X,\theta \right)\right). $$

The problem can be solved numerically using the Gauss–Newton algorithm, and standard errors are computed using a wild bootstrap procedure [18].

### Nonparametric model

Consistent parametric estimation of the elasticities of capital and labor relies on the assumption that the parametric functional form chosen, in many cases the Cobb-Douglas, is the correct or true functional form. However, there is no consensus in the health economics literature regarding the correct functional form for the delivery of primary health care. Nonparametric estimation of function (1) allows us to avoid the bias of incorrectly imposing a certain parametric shape to the relationship between inputs *K* and *L* and output *Y*. In fact, one of the main advantages of the nonparametric approach is that it recovers the relationship between inputs and output directly from the data.

We use a Local-Linear Least Squares model (LLLS) to approximate the function *f*(*K*, *L*) in eq. (1).^2^ The LLLS estimator is perhaps the most popular nonparametric regression estimator. To simplify notation, let *X* denote the input matrix (*K*, *L*). The LLLS estimator fits a line on the neighborhood of a point *X*_0_, where the concept of “neighborhood” is determined by a bandwidth vector *h*. The estimator re-writes the original model by considering a Taylor approximation around *X*_0_:

$$ Y=f\left({X}_0\right)+\left(X-{X}_0\right)\beta \left({X}_0\right)+\varepsilon, $$

where *β* is the gradient and is treated like a parameter to be estimated, and so is *f*(*X*_0_). Denoting *a* = *f*(*X*_0_) and *b* = *β*, the LLLS chooses *θ* = (*a*, *b*) to minimize the weighted sum of squared residuals:

$$ \underset{\theta }{\min}\left(Y-\overset{\sim }{X}\theta \right)^{\prime }K\left(X,{X}_0,h\right)\left(Y-\overset{\sim }{X}\theta \right), $$

where $\overset{\sim }{X}=\left(1,X-{X}_0\right)$, 1 is a column vector of ones and $K\left(X,{X}_0,h\right)=\frac{1}{\sqrt{2\pi }}{e^{-\left(\frac{1}{2}\right)\left(\frac{X-{X}_0}{h}\right)}}^2$ is the Gaussian Kernel. When the Kernel is the identity matrix, the estimator reduces to the OLS estimator. By using Kernel weights we mitigate the effect of poor approximations of points far from *X*_0_. Standard errors and confidence intervals can be computed using a wild bootstrap. To avoid issues with numerical optimization and facilitate estimation, we standardize the data by dividing each variable by their mean.

The final step required to implement the above LLLS nonparametric estimator is to choose bandwidths in *h*. Typically, the literature relies on data-driven methods to determine the appropriate set *h* [18]. We use least squares cross validation (LSCV) to determine the bandwidths in *h*. The idea is simple and relies on choosing *h* that minimizes the sum of the squared errors of the model prediction, which in turn depends on *h*. Formally, the bandwidths in *h* minimize $\sum {\left(Y-\hat{Y}(h)\right)}^2$.

In summary, the concept of the LLLS is to fit a linear model around the neighborhood of inputs *K* and *L*, where this neighborhood is determined by a bandwidth chosen using cross validation (LSCV). The nonparametric model moves along the distributions of *K* and *L* estimating local linear regressions, connecting the predicted outputs (or conditional mean) from these various regressions to generate the relationship between inputs and output.

The nonparametric estimates of the elasticities of capital and labor are computed as.

$$ \hat{\beta}(K)K/\hat{Y}\ \mathrm{and}\ \hat{\beta}(L)\ L/\hat{Y} $$

respectively, where $\hat{\beta}(K)$ is the gradient of the conditional mean with respect to capital, $\hat{\beta}(L)$ is the gradient of the conditional mean with respect to labor, and $\hat{Y}$ is the fitted value. Standard errors for elasticities, returns to scale, the predictions are computed via wild bootstrap as it is consistent under both homoskedasticity and heteroskedasticity [18]. Estimation was done using the R software [22] using codes provided by Henderson and Parmeter [18].

### Data

Our analysis is based on a sample of SUS users from the 100 largest cities in Brazil. The data contains information about SUS medical care delivery, physical medical infrastructure, and supply of healthcare professionals, at the city-level, monthly, from 2012 to 2016. Preliminary examination of the data revealed that the two largest cities (São Paulo and Rio de Janeiro) represent extreme values when compared against the remainder 98. We therefore exclude these two cities from our analysis. Our working sample offers a fair representation of urban Brazil. Collectively, the 98 cities considered in this study account for approximately one third of Brazil’s total population.

The data comes from DATASUS – SUS IT department. DATASUS has several SUS databases that are publicly available for download.^3^ Our output data were collected from the SIA database -- System of Outpatient Information (Sistema de Informação Ambulatorial – SIA). We measure primary care output as the number of doctor’s visits in the SUS system, for each city-month observation. The number of patient visits (per unit of time) is a typical measure of primary care output [11]. Our input data are from the CNES database -- National Registry of Healthcare Facilities (Cadastro Nacional de Estabelecimentos de Saúde – CNES). Our proxy for health capital infrastructure is the city’s number of clinics and similar health care delivery units. The city’s stock of labor is measured as the number of physicians working in the city’s SUS system.^4^

**Fig. 1:**
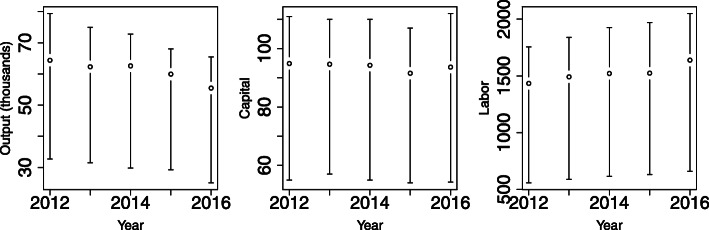
Mean and IQR of output, capital, and labor, by year

In the 98 cities considered in this study, from 2012 to 2016, the sample contains 274,175 distinct physicians, 11,203 distinct clinics, and 407,259,570 primary care consultations. Figure 1 shows the (city-month) average number of consultations, clinics, and doctors, along with the interquartile range (IQR), by year. The data show that the representative city-month observation hovers around 60 thousand consultations (with a slight decline from 2012 to 2016), just under 100 clinics (steady trend in the period), and approximately 1500 doctors (with a slight upward trend).

## Results

We start by reporting the elasticities of capital and labor estimated by the parametric model. The simplicity of the Cobb-Douglas model is a feature that makes such a parametrization attractive and contributes to the popularity of this model. On the downside, the Cobb-Douglas model does not allow the elasticities to vary along different levels of capital and labor. The model’s functional form leads to a single elasticity measure for each input, and therefore does not account for nonlinearities and cross-effects.^5^

Table 2 shows the Cobb-Douglas elasticities estimates along with their standard errors. We find elasticities of output with respect to capital and labor of similar magnitude. The elasticities are precisely estimated, as indicated by the narrow standard errors. Both inputs have elasticity around 0.38, which suggests diminishing returns to scale in the order of 0.76. As a result, we find that primary care delivery increases by less than the proportional increase in both medical capital infrastructure and labor requirements. The Cobb-Douglas results suggest that if policy makers double capital and labor, primary care output will less than double and increase by a factor of 1.5.

**Table 2:** Estimates of the Cobb-Douglas model

Capital Elasticity	0.378
	(0.016)
Labor Elasticity	0.381
	(0.015)
Returns to Scale	0.759
	(0.013)

Bootstrapped standard errors are in parenthesis (based on 400 replications)

As discussed above, consistency of parametric results relies on strong assumptions. To be unbiased, the Cobb-Douglas functional form must be the appropriate functional form for primary health care production. That would imply constant elasticities, and there exists no evidence that this is the case in primary care delivery.

Next, we use nonparametric models to obtain estimates that are not plagued by parametric misspecifications. The elasticities estimates are driven by the data and are allowed to vary along the distribution of capital and labor. As a result, the nonparametric approach captures nonlinear effects allowing policy makers to have a deeper understanding of the heterogeneity in returns to scale. It is possible, for example, to capture diminishing returns to scale in some input range and increasing returns to scale in another.

We estimate a nonparametric LLLS model using LSCV to select bandwidths. Table 3 reports the bandwidth estimates. We find bandwidths of 0.037 and 0.453, for capital and labor, respectively. For both values, the estimates are significantly lower than the upper bound suggested in the literature of two times the standard deviation of the corresponding input [23]. This indicates that both capital and labor have a nonlinear effect on output.

**Table 3:** LSCV Bandwidths

	Bandwidth	Upper Bound
Capital	0.037	1.881
Labor	0.453	3.716

The nonparametric model produces observation-specific elasticities. Table 4 summarizes results by showing the LLLS elasticities estimates at their mean, 25th quantile, median, and 75th quantile. The table reveals several important lessons. First, focusing at the mean, while the nonparametric estimate of the elasticity of capital is similar to that of the parametric model, the LLLS estimate of the elasticity of labor is significantly smaller than the Cobb-Douglas counterpart. Table 4 shows that mean returns to capital (0.391) are more than seven times larger than mean returns to labor (0.050). That has a significant effect on the nonparametric estimates of returns to scale. The nonparametric model estimates diminishing returns to scale of 0.441, i.e. returns diminishing stronger than those of the parametric model. These estimates suggest that doubling input usage leads to an increase in output of a factor of 0.88 (as opposed to 1.5 in the Cobb-Douglas model).

**Table 4:** Results of the nonparametric model

	Mean	Quantile of Elasticity Distribution		
		0.25	0.50	0.75
Capital Elasticity	0.391	−0.903	0.281	1.174
	(0.246)	(0.260)	(0.166)	(0.678)
Labor Elasticity	0.050	−0.227	0.096	0.380
	(0.077)	(0.062)	(0.122)	(0.136)
Returns to Scale	0.441	−0.901	0.532	1.376
	(0.278)	(0.177)	(0.351)	(0.246)

Bootstrapped standard errors are in parenthesis (based on 400 replications)

Next, we find significant heterogeneity on both sets of elasticities. In the 25th quantile (i.e. the bottom of the distribution), the elasticities of both capital and labor are negative and represent diseconomies of scale. This is an indication of the existence of dysfunctional local health systems where investments in medical inputs actually drive patients away. Diseconomies of scale in the healthcare sector have been reported both in developed countries, e.g. the United States [24], and in developing countries, e.g. Turkey [13]. At the median, returns to capital are 28% smaller than the mean, and returns to labor are almost doubled.

Finally, elasticity heterogeneity becomes even more evident when we examine the top of the distribution. In the 75th quantile, the elasticity of output with respect to capital is 1.174, which suggests increasing returns to capital. Labor continues to exhibit diminishing returns. Comparing parametric and nonparametric estimates, only at the 75th quantile the LLLS estimate of the elasticity of labor is similar to that of the Cobb-Douglas model. In general, the 75th quantile results indicate that urban Brazil has high functioning local health care systems with strong increasing returns to scale.

When examining production functions, another interesting way to present nonparametric findings is to plot the estimate of the conditional mean of output (and its confidence interval) against one input. When production depends on two inputs, as in our case, this plot needs to be constructed by holding the other input constant at some (arbitrary) level [18]. The exercise we pursue in this paper is to plot output estimates obtained using one variable input (capital or labor), holding the other input fixed. For each case (variable capital or variable labor), we estimate three models that differ by the level of the fixed input (which is fixed at 25th, 50th, and 75th quantiles). This examination allows us to uncover cross-effects and assess whether there exist complementarities between the medical capital infrastructure and the supply of physicians.

**Fig. 2:**
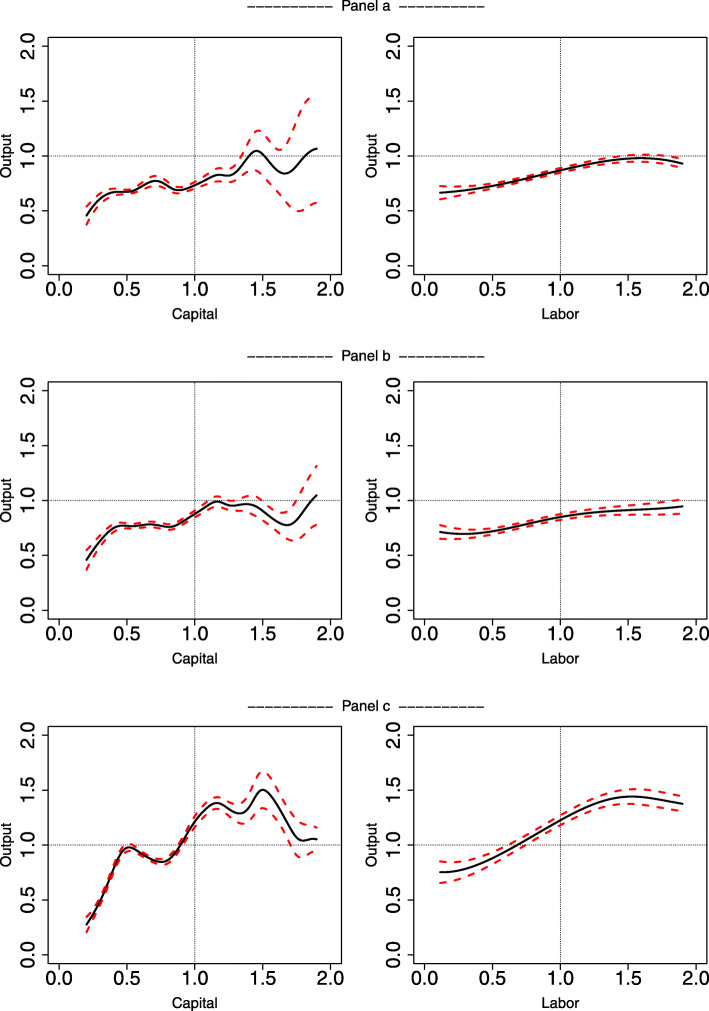
Conditional mean output estimates versus a single input. PANEL **a** – Prediction holds the other input fixed at the 0.25 quantile. PANEL **b** -- Prediction holds the other input fixed at the median. PANEL **c** – Prediction holds the other input fixed at the 0.75 quantile

Results are shown in Fig. 2. The graphs in left column show the counterfactual exercise of displaying output prediction as a function of capital, holding labor fixed at the 25th quantile (PANEL a), 50th quantile (PANEL b), and 75th quantile (PANEL c). The right column displays the same exercise for varying labor. We normalize output and inputs by their mean to avoid issues with numerical optimizations. As a result, an output (input) value of 1 represents mean output (input). Similarly, a value of 2 represents a level twice the mean. In addition to facilitate optimization, this normalization simplifies the scale of the graphs, making them easier to read while retaining economic meaning by allowing for cross comparisons.

The counterfactual plots of output against capital (holding labor constant) are far more volatile than the ones of output and labor (holding capital constant). This finding suggests that there is more uncertainty in health care capital investments than in investments to expand the number of physicians. In general, as input usage increases, output increases. But there are downward-sloping regions, especially on the left-hand side graphs for high levels of capital. The right-hand side column shows a similar effect for labor, however with significant less variance and a shallower negative output response at the upper tail of the labor distribution.

The counterfactual analysis complements the previously discussed elasticity analysis. The graphs allow us to gauge the slope of the production function at different points, which shows information about the general regions with diseconomies of scale, diminishing returns, and increasing returns. While results in Table 4 show that there are negative elasticities in the bottom of the elasticity distribution (25th quantile), Fig. 2 suggests that many of these negative results occur at the top of both the capital and labor distributions.

In Fig. 2, a comparison of rows within a column reveals complementarities between capital and labor, but these are not uniform. For instance, on the right-hand side column, in PANEL a, the entire prediction of output lies below the mean output (or below 1). The behavior of the conditional mean as labor varies does not change much when capital increases from the 25th quantile to the median (i.e. comparing right-hand sides of PANELS a and b). One exception is that negative returns to labor at the top of the labor distribution disappear when capital increases from 25th to 50th quantile. However, moving down to PANEL c (i.e. output estimates hold capital fixed at the 75th quantile), we see that output is at a higher level and many points are above the mean. Moreover, output response to labor is steeper when capital level is high. In general, we conclude that medical infrastructure complements physicians mainly when capital levels are high, but high capital levels may also induce diseconomies of scale for expansion of physicians at the top of the labor distribution.

## Discussion

We estimate a primary care production function using data from the public health care systems of the largest cities in Brazil. In doing so, we compare the insights from the popular Cobb-Douglas parametric model against those from a nonparametric model. Our results highlight the rich set of policy-related information that can be generated by flexible nonparametric estimates. With the fast-pace development of information systems making large datasets available to managers, the application of nonparametric methods offers flexibility in an environment where the standard “curse of dimensionality” can be circumvented.

Nevertheless, the study has limitations. While the nonparametric model is able to deliver a variety of interesting insights by exploring the heterogeneity in the data, in our application for primary care in urban Brazil, we find estimates that are less efficient than those from the parametric model. For instance, while Table 3 reports a parametric elasticity of output to capital of 0.378 with standard error of 0.016, Table 4 reports a similar nonparametric mean elasticity of capital of 0.391, however, with much larger standard error of 0.246.

The paper’s output is the city’s number of primary care medical consultations. Although it is not always necessarily the case that efforts to increase the number of consultations are needed or desired, it seems reasonable to expect that increases on primary care consultations in urban Brazil are welfare enhancing. In our sample, the average number of primary care consultations per person per year is 0.4, which is significantly below the SUS recommendation of 2–3 visits/person/year [25].

While the approaches presented in the paper are applied to local health care systems (i.e. cities), the measurement of health care production and its associated efficiency can also be applied at the health care delivery unit level. This method would require output and input data at the hospital or clinic level. Such an approach would offer micro-level information that could inform budgetary decisions. Here, a word of caution is warranted. The direct application of production and efficiency estimates to reimbursement frameworks can be problematic as production models are limited by their ability to capture nuances of the health care delivery process. For example, if output measures are not adjusted for quality of care, the models may underestimate the return to investments and high-quality health care facilities could appear to be unproductive [26].

Adopting a more aggregated perspective that examines local healthcare systems, our models estimate elasticities and identify the existence of significant heterogeneities in urban Brazil. Our approach is able to identify that some local healthcare systems function well, while others do not. However, our approach does not inform the drivers of these results, nor the reasons and specific mechanisms underlying undesirable outcomes. In that, this study represents only the first step of a more comprehensive analysis of primary care. Our methods are able to identify cities with high-return health systems that may represent investment opportunities, and cities with diseconomies of scale whose local healthcare sector should be examined more closely. Future research is needed to investigate the role of characteristics of the city, such as sociodemographic composition (e.g. age, education, income), on determining the returns to scale in health care.

## Conclusions

Many countries struggle with fiscal challenges and must overcome great difficulties to finance health care systems. This highlights the growing importance of efficient investments in primary health care, especially in fiscally challenged countries with large health care systems like Brazil. It is, therefore, fundamental to identify investments opportunities to leverage budgets and achieve maximum outcomes.

Flexible nonparametric production methods can help design health care policy. For the most part, the nonparametric literature applied to health care production has focused on envelopment techniques such as Data Envelopment Analysis (DEA) and Free Disposal Hull (FDH) to estimate a health care production frontier [10, 27–32]. However, envelopment estimators are sensitive to outliers and extreme values and, as a result, may deliver biased estimates of returns to scales, which compromises the ability to inform health care investments [33, 34].

This study shows how nonparametric methods can be used to inform public health policy. We estimate a LLLS production model that is, by construction, less sensitive to extreme values and outliers than envelopment estimators. The model is applied to a large dataset of Brazilian cities with more than 400 million consultations, 270 thousand physicians, and 11 thousand clinics, from 2012 to 2016.

We find that, while the results of the typically used CD parametric model suggest that average returns to medical capital and labor in urban Brazil are similar, the more flexible nonparametric estimates indicate that average returns to capital are almost 8 times larger than returns to labor. That is, capital investments promote, on average, higher uptake of primary care services. In order words, nonparametric results suggest that, on average, expanding the number of clinics, holding the number of physicians constant, is more effective than expanding the number of physicians, holding the number of clinics constant.

The nonparametric model allows us to go beyond average estimates and explore the heterogeneity in returns to capital and labor. We find that when the goal is to increase the uptake of primary care services in Brazil, investments in health care capital are more uncertain than expanding the number of physicians. Results reveal significant heterogeneity on returns to scale along the distribution of medical capital and labor, however, returns to capital infrastructure are generally higher than returns to physicians. Medical infrastructure complements physicians mainly when capital levels are high, but high capital levels may also induce diseconomies of scale when expanding the number of physicians at the top of the labor distribution.

A recent World Health Organization report shows that global health spending is on an upward trajectory [35]. This is especially important for Brazil. During the period 2012–2016, Brazil’s annual health spending averages $1325 (international dollars) per capita, which ranks Brazil as the 58th country in health spending, globally. This level of investment represents only 28% of that from OECD countries, or 52% of the investments of Europe and Central Asia [2]. These statistics are striking considering that Brazil was the 7th largest economy in the world in 2012 (behind only U.S., China, Japan, Germany, U.K., and France). In fact, despite having economies of similar size, Brazil’s health expenditure (per capita) is only 29% that of France.

Currently, the Brazilian federal government faces a tremendous amount of political and popular pressure to increase health investments and expand the SUS network. In a scenario of health expansion, understanding nonlinearities and complementarities between medical inputs is important as it allows policy makers to predict the returns of increasing the availability of an input, depending on the profile of each local market (e.g. scale and level of input complementarity). This information can be harnessed to determine, for instance, priority regions where investments have higher productivity.

## Supplementary Information

**Additional file 1:**

## Data Availability

The datasets generated and/or analysed during the current study are available in the DATASUS website: datasus.saude.gov.br/informacoes-de-saude-tabnet .

## Abbreviations

CD: Cobb-Douglas
CNES: National Registry of Healthcare Facilities
DATASUS: SUS IT department
DEA: Data Envelopment Analysis
FDH: Free Disposal Hull
GDP: Gross Domestic Product
IQR: Interquartile Range
LLLS: Local-Linear Least Squares
LSCV: Least Squares Cross Validation
NLS: Nonlinear Least Squares
OLS: Ordinary Least Squares
SIA: System of Outpatient Information
SUS: Brazilian Unified Health System
WHO: World Health Organization

## Footnotes

1 Henderson and Parmeter [18], in Chapter 5, offer further details and a comprehensive discussion of alternative parametric (Constant Elasticity of Substitution, Generalized Quadratic, and Generalized Leontief) and nonparametric (Local-Constant and Local-Polynomial) models.

2 Refer to Henderson & Parmeter for a detailed discussion of the LLLS estimator [18].

3 SUS data are available online at datasus.saude.gov.br/informacoes-de-saude-tabnet.

4 Refer to Electronic Supplemental Material for further details on measurements of capital, labor, and output.

5 It can be easily verified that $\left(\frac{\partial A{K}^{\alpha }{L}^{\beta }}{\partial K}\right)\left(\frac{K}{A{K}^{\alpha }{L}^{\beta }}\right)=\alpha$ and $\left(\frac{\partial A{K}^{\alpha }{L}^{\beta }}{\partial L}\right)\left(\frac{L}{A{K}^{\alpha }{L}^{\beta }}\right)=\beta$.
