# Supplementary material for: Nonparametric estimation of a primary care production function in urban Brazil
Source: Health Econ Rev. 2020 Nov 28;10:37. doi: 10.1186/s13561-020-00294-9 (PMC7700717; doi:10.1186/s13561-020-00294-9)
Supplement: Supplementary file 1 — Additional file 1: [file 13561_2020_294_MOESM1_ESM.docx]

**Electronic Supplementary Material**

Nonparametric Estimation of a Primary Care Production Function in Urban Brazil

Bruno Wichmann and Roberta Wichmann

**Measuring Primary Care Output – Doctor’s visits**

Our output measure is the city-month aggregation of the number of patients visits associated with primary care procedures delivered by SUS, from Jan 2012 to Dec 2016. The data were collected from the PA (Produção Ambulatorial) files in the SIA database. The types of visits/procedures considered are listed in Table S1, along with their SIA codes. These procedures represent the main primary care services offered by SUS.

**Table S1: SIA procedure codes and description**

| **Procedure Code** | **Description** |
| --- | --- |
| 301010013 | Doctor visit – consultation with patient cured of Tuberculosis |
| 301010021 | Doctor visit – consultation with patient diagnosed with Tuberculosis |
| 301010030 | Primary care consultation (with other healthcare professionals) |
| 301010064 | Doctor visit – primary care consultation |
| 301010080 | Doctor visit – puericulture consultation |
| 301010099 | Doctor visit – clinical evaluation of smokers |
| 301010110 | Doctor visit – prenatal consultation |
| 301010129 | Doctor visit – puericulture consultation |
| 301010137 | Home medical consultation |
| 301010056 | Medical consultation – worker health |
| 301040028 | Clinical care – consultation or insertion of intrauterine device |
| 301040087 | Primary care group consultations |
| 301050090 | Medical consultation – diagnosis of death |
| 301060037 | Urgent care in primary care |
| 301060045 | Urgent care in primary care (observation care up to 8 hours) |
| 301060053 | Urgent care in primary care (followed by patient transfer) |
| 301080011 | Cognitive behavioral therapy - smoking |
| 413010023 | Urgent care – small burns |

**Measuring Capital – Number of Clinics**

Our proxy variable for capital is the number of all primary care delivery facilities (excluding hospitals and mobile units) operating in a city in the month of reference. The data was collected from the ST (Estabelecimentos) files in the CNES database. Table S2 shows the list of the types of facilities considered in this study, along with their respective CNES codes. In general, these facilities are different types of medical clinics.

**Table S2: CNES type of facility -- codes and description**

| **Unit Type Code** | **Description** |
| --- | --- |
| 01 | Medical clinic |
| 02 | Health center |
| 04 | Polyclinic |
| 22 | Single doctor’s office |
| 36 | Speciality clinic |
| 50 | Health surveillance unit |
| 61 | Birthing center |
| 64 | Health services regulation center |
| 68 | Municipal health secretariat |
| 71 | Health support center – “Saúde da Família” |
| 72 | Indigenous people’s health unit |
| 73 | Urgent care units in primary care |

**Measuring Labor – Number of Physicians**

Our measure of healthcare labor is the total number of doctors in the SUS system, by city-month, from Jan 2012 to Dec 2016. The data was collected from the PF (Profissionais) files in the CNES database. We used four occupation codes to identify medical doctors: 2231, 2251, 2252, and 2253 (based on the Brazilian classification of occupations – CBO, Ministry of Labor).^[[1]](#footnote-1)^ We only considered doctors that reported a positive number of hours worked in the SUS system, in the month of reference.

1. For details refer to the Government of Brazil, Ministry of Labor, at https://empregabrasil.mte.gov.br/76/cbo/. [↑](#footnote-ref-1)
